# Malaria in pregnancy (MiP) studies assessing the clinical performance of highly sensitive rapid diagnostic tests (HS-RDT) for *Plasmodium falciparum* detection

**DOI:** 10.1186/s12936-023-04445-1

**Published:** 2023-02-20

**Authors:** Xavier C. Ding, Sandra Incardona, Elisa Serra-Casas, Sarah C. Charnaud, Hannah C. Slater, Gonzalo J. Domingo, Emily R. Adams, Feiko O. ter Kuile, Aaron M. Samuels, Simon Kariuki, Sabine Dittrich

**Affiliations:** 1 FIND, Geneva, Switzerland; 2 Diagnostics Program, PATH, Seattle, USA; 3 Department of Tropical Disease Biology and Department of Clinical Sciences, Liverpool School of Tropical Medicine, Liverpool, L3 5QA UK; 4 Malaria Branch, Division of Parasitic Diseases and Malaria, Center for Global Health, Centers for Disease Control and Prevention (CDC), Kisumu, Kenya; 5 Malaria Branch, Division of Parasitic Diseases and Malaria, Center for Global Health, Centers for Disease Control and Prevention (CDC), Atlanta, Georgia USA; 6 Kenya Medical Research Institute-Centre for Global Health Research, Kisumu, Kenya

**Keywords:** Malaria, Pregnancy, Rapid diagnostic test, RDT, Highly sensitive rapid diagnostic test, HS-RDT, uRDT

## Abstract

**Background:**

Rapid diagnostic tests (RDTs) are effective tools to diagnose and inform the treatment of malaria in adults and children. The recent development of a highly sensitive rapid diagnostic test (HS-RDT) for *Plasmodium falciparum* has prompted questions over whether it could improve the diagnosis of malaria in pregnancy and pregnancy outcomes in malaria endemic areas.

**Methods:**

This landscape review collates studies addressing the clinical performance of the HS-RDT. Thirteen studies were identified comparing the HS-RDT and conventional RDT (co-RDT) to molecular methods to detect malaria in pregnancy. Using data from five completed studies, the association of epidemiological and pregnancy-related factors on the sensitivity of HS-RDT, and comparisons with co-RDT were investigated. The studies were conducted in 4 countries over a range of transmission intensities in largely asymptomatic women.

**Results:**

Sensitivity of both RDTs varied widely (HS-RDT range 19.6 to 85.7%, co-RDT range 22.8 to 82.8% compared to molecular testing) yet HS-RDT detected individuals with similar parasite densities across all the studies including different geographies and transmission areas [geometric mean parasitaemia around 100 parasites per µL (p/µL)]. HS-RDTs were capable of detecting low-density parasitaemias and in one study detected around 30% of infections with parasite densities of 0–2 p/µL compared to the co-RDT in the same study which detected around 15%.

**Conclusion:**

The HS-RDT has a slightly higher analytical sensitivity to detect malaria infections in pregnancy than co-RDT but this mostly translates to only fractional and not statistically significant improvement in clinical performance by gravidity, trimester, geography or transmission intensity. The analysis presented here highlights the need for larger and more studies to evaluate incremental improvements in RDTs. The HS-RDT could be used in any situation where co-RDT are currently used for *P. falciparum* diagnosis, if storage conditions can be adhered to.

**Supplementary Information:**

The online version contains supplementary material available at 10.1186/s12936-023-04445-1.

## Background

Malaria in pregnancy (MiP) is associated with increased risk of poor maternal and infant health outcomes, including fetal loss, maternal anaemia, pre-term birth, low birthweight and intrauterine growth retardation, which in turn increase the risk of infant morbidity and mortality [1]. Modelled estimates indicate that in 2019, 35% of pregnancies in sub-Saharan Africa (or 11.6 million expectant mothers) were exposed to malaria infections, leading to 0.8 million low birthweight newborns [2, 3]. Malaria prevalence is highest in women who are primigravid and who are in the first or second trimester of pregnancy [4]. To protect against MiP, a strategy of routine intermittent preventative treatment during pregnancy (IPTp) with sulfadoxine-pyrimethamine (SP) is recommended at each scheduled antenatal care visit in the second and third trimester, at a minimum of monthly intervals [5]. However, IPTp with SP is contraindicated in the first trimester of pregnancy, is threatened by parasite resistance to SP, is only recommended in moderate and high transmission areas of sub-Saharan Africa, and the uptake is poor [3], limiting the effectiveness of this intervention.

WHO has reviewed different ‘Test and Treat’ strategies in the context of MiP, such as Intermittent Screening and Treatment (ISTp) at the first antenatal care (ANC) visit [6]. Although wide-scale deployment of rapid diagnostic tests (RDTs) for the detection of malaria has been largely successful in control efforts in the general population, pooled analyses have shown that interventions based on maternal screening with conventional RDTs (co-RDT) did not provide any additional benefit as compared to routine IPTp-SP [6, 7].

IPTp is contra-indicated in the first trimester of pregnancy, yet a high proportion of women are infected in the first trimester [8], which can have devasting consequences for the pregnancy [9–11]. Early detection and treatment of infections is, therefore, essential. However, detection is difficult as pregnant women infected with *Plasmodium falciparum* are commonly asymptomatic and have low density infections [12], and infected erythrocytes that sequester in the placenta escape detection in peripheral blood [9]. An important proportion of these low-density malaria infections remain undetected by light microscopy or co-RDTs [13]. A modelling study on malaria screening strategies at ANC visits suggested that more sensitive RDTs could provide incremental improvements in ISTp strategies over IPTp-SP in terms of reducing infection prevalence at delivery, with presumably better outcomes for mother and child [8]. It also highlighted that ISTp during the first trimester could improve pregnancy outcomes [8]. Improved point-of-care diagnostics that can detect more cases of maternal infections and in particular placental malaria may thus contribute to a reduction of adverse clinical outcomes for mothers and newborns in malaria endemic settings.

A highly sensitive RDT (HS-RDT) based on detection of Histidine-Rich Protein 2 (HRP2) was developed (NxTek™ Eliminate malaria Pf, Abbott Diagnostics) to improve the identification of *P. falciparum* infections below the detection limit of co-RDTs. The HS-RDTs enable point-of-care testing with an analytical sensitivity (limit of detection (LOD)) that is ten-fold lower than that reported for current best-in-class co-RDTs, at 80 pg of HRP2 per mL of blood [14] compared to 800-1000 pg for co-RDTs [15] as tested in vitro in malaria-negative blood. A recent review of data across multiple studies in different settings show that this improvement in analytical performance consistently results in an improvement in sensitivity and prevalence estimates as compared to PCR reference testing. The significance of this improvement varies from setting to setting [16–18].

In June 2018, the WHO Global Malaria Programme convened a technical consultation to identify the evidence required to develop recommendations on the use of the HS-RDT in different contexts including the detection of MiP [19]. The lack of results from prospective longitudinal studies or trials assessing the clinical impact of the HS-RDT as part of interventions to prevent MiP was noted. It was concluded that diagnostic accuracy studies comparing the HS-RDT to co-RDT are of high priority, and that these should reflect a range of different conditions (*e.g.* transmission intensities, target populations). This review compiles all existing evidence to date on the performance and use case scenarios of the HS-RDT in the context of pregnancy to inform policy makers and the research community on existing evidence and knowledge gaps.

## Methods

This evidence review encompassed both published articles in peer-reviewed journals as well as other grey literature such as technical reports or presentations at scientific conferences.

### Collection of data through online resources

Information was gathered on completed and ongoing research projects or grants reported between January 2017 to December 2020 in the following databases: MEDLINE–Pubmed Central, Malaria in Pregnancy Library (Malaria in Pregnancy Consortium), Cochrane Library, MESA Track database, Conference books (MIM, ECTMIH, ASTMH), ClinicalTrials.gov, WHO–International Clinical Trials Registry Platform, ICH GCP–Clinical Trials Registry, Grantome.com, Europe PMC Grant Finder, National Institutes of Health (NIH). The keywords used for the project search were: malaria, pregnant/pregnancy, RDT, diagnostic, (highly/ultra)-sensitive, HS-RDT, hsRDT, uRDT, Alere.

### Contact with experts and stakeholders

Experts and stakeholders were contacted to further identify ongoing studies. This included 25 active researchers in the field of MiP or malaria diagnostics, as well as focal persons in international organizations working in these fields. Each contact was also asked to name other experts in the field who were then contacted. All Principal Investigators of identified eligible studies were contacted in order to confirm their study details and the accuracy of the data included in the comparative analyses presented in this review.

### Study inclusion criteria

Prospective studies of any design were eligible if they included pregnant women during pregnancy (any trimester) or at delivery, used the NxTek™ Eliminate malaria Pf RDT by Abbott Diagnostics (formerly the Alere Malaria Ag Pf (05FK140)), and reported *P. falciparum* detection using any reference assay.

### Analysis of methodology

The product disclosure statements were assessed for all co-RDTs used in the trials and all were assessed to be of high quality. All studies used the recommended 5 µL of blood to perform the RDT. The molecular reference standards used in the completed studies were quantitative PCR (qPCR; 3), quantitative Reverse Transcription PCR (qRT–PCR; 2), Photo-induced-electron-transfer PCR (PET–PCR; 1), nested PCR (nPCR; 1) and a composite qPCR + Loop-mediated isothermal DNA amplification (LAMP; 1). The equivalent volume of blood from which DNA was extracted and used for the molecular methods varied and was not known for most studies, however the Benin study used 1.7 µL blood equivalent. LOD or limit of quantification (LOQ) were used as determined and reported in the study where possible, or based on the reference for the technique.

### Data analysis

Quantitative and qualitative data from all the eligible studies were collected in multiple Excel spreadsheets. Figures were prepared with Graphpad Prism 8.1. (GraphPad Software, Inc., CA, US), and additional test performance analyses (i.e. not provided in published articles) of Pearson and Spearman correlations and paired t-test were conducted with STATA version 11 software (StataCorp LP, College Station, TX, USA). Efforts were made to perform meta-analyses, but the heterogeneity of the studies was too great to provide meaningful results.

## Results

### Eligible studies

A total of 13 studies addressing HS-RDT use in pregnancy were identified as of December 2020 (Table 1). Eight studies were completed by this date. A further three studies had completed field activities, but the data were not available for this review, and five studies were ongoing.

**Table 1:** Study design characteristics of the identified studies evaluating the use of the HS-RDT for the detection of MiP

No.	Country	First Author or PI in clinical trial record	Period	Malaria transmission	Objective of HS-RDT evaluation		Study design	Participants				HS-RDT samples				Institution and References
					Clinical performance (test accuracy)	Clinical impact (benefits and applications		Asymptomatic	Symptomatic	Sample size	Mol+ Sample	Peripheral	Placental	Specimen type	Testing context	
*Completed and analyzed*																
1	Benin	V. Briand	Dec 2013–Jul 2017	High	Y based on PCR	Y Association HS-RDT positivity and clinical outcomes	Preconceptional cohort, recruitment at community level. HS-RDT testing at 1st/3rd trimester ANC visit and at delivery.	X	X	942*	172	X	X	Thawed blood	Research Lab	IRD, FIND [22]
2	Colombia (1)	A.M. Vasquez	Sept–Dec 2017	Low	Y based on PCR	N	Observational cross-sectional trial. Retrospective study. ANC visits or at delivery.	X	X	737	35	X	X	Thawed blood	Research Lab	Univ. of Antioquia, FIND [26]
3	Colombia (2)	A.M. Vasquez	May 2017–Jan 2018	Low	Y based on PCR	N	Observational cross-sectional trial. ANC visits.	X	X	858	39	X		Fresh blood	POC (ANC clinic)	Univ. of Antioquia, FIND [27]
4	Indonesia	V.T. Unwin	Mar–Jun 2018	Moderate	Y based on PCR+LAMP (composite)	N	ANC visits (participants of a MiP intervention study)	X		270	158	X		Thawed red blood cells + plasma	Research Lab	LSTM [23] Parent study [24]
5	Kenya (1)	A.M. Samuels	April–Sept 2018	High	Y – [a]based on PCR	Y – [b]Correlation HS-RDT-positivity in ANC and Malaria Indicator Surveys (as proxy for surveillance)	[a]: First ANC visit.[b]: Community based Malaria Indicator Survey (cMIS) + all ANC visits.	X	X	[a]: 482 [b]: 4000/y	172	X		Fresh blood	POC (ANC clinic)	LSTM, CDC, KEMRI [25]
*Completed (analysis ongoing)*																
6	Papua New Guinea	L. Robinson	Jun–Dec 2018	Low	Y based on PCR	N	Observational cross-sectional trial. ANC visits.	X	X	918	X			Fresh blood	POC (ANC clinic)	Burnet Institute, PNG IMR, FIND [36]
7	Kenya (2)	E.R. Adams,F.O. ter Kuile	Oct 2018–May 2019	High	Y based on PCR	N	ANC visits (participants of a MiP intervention study)	X		493	X			Fresh blood	POC (ANC clinic)	PI E Adams, F ter Kuile Parent study [37]
8	Malawi	D.P. Mathanga, J. Gutman	Jan 2017–Dec 2019	Moderate/high	Y based on PCR	N	Population cross-sectional survey: cohort followed from first ANC visit to delivery (participants of a MiP intervention study). HS-RDT testing at delivery.	X	X	601	X		X	Fresh blood	Field Lab	Univ. of Malawi, CDC [38] Parent study [39]
*Ongoing studies*																
											Diagnostics (in addition to HS-RDT)				Testing context	Ref
										PCR	LAMP		Co-RDT	LM		
9	Burkina Faso (1)	Tahita M.C,Tinto H.	Aug 2020–Feb 2022	High	N	Y Operational feasibility and impact of additional screening with HS-RDTs	ANC visits (16–24 weeks at their first booking)	X	X	qPCR (if budget available)	–		Yes	Yes	POC (ANC clinic)	Institut de Recherche en Sciences de la Santé/Clinical Research Unit of Nanoro (CRUN) [31]
10	Burkina Faso (2)	Tahita M.C,Tinto H.	Dec 2020–May 2021	High	Y based on PCRand microscopy	N	ANC visits (16–24 weeks at their first booking)	X	X	qPCR	–		Yes	Yes	Lab conditions	Institut de Recherche en Sciences de la Santé/Clinical Research Unit of Nanoro (CRUN)
11	Senegal	Programme National de Lutte Contre le Paludisme (PNLP)	Dec 2019–TBC				ANC visits							Yes	POC (ANC clinic)	Programme National de Lutte Contre le Paludisme (PNLP)
12	Nigeria	W. Oyibo	Jan–April 2021	Moderate/high	Y Compared to co-RDT and LM		ANC visits.			DBS collected, but not planned in short term	–		YesPf/Pan	Yes	POC (ANC clinic)	University of Lagos PI W Oyibo
13	DRC	H Muhindo, V Maketa, H Schallig, PF Mens, K Kayentao	Dec 2020–Dec 2022	High	Y Compared to qPCR	N	ANC visits and at time of delivery	X	X	YesqPCR	–		-	Yes	POC (ANC clinic)	University of KinshasaAcademic Medical Centre (Amsterdam)Malaria Research and Training Center (Bamako) [40]

*IRD* Institut de Recherche pour le Développement; *FIND* Foundation for Innovative New Diagnostics; *CDC* Centre for Disease Control; *KEMRI* Kenya Medical Research Institute; *PNG* *IMR* Papua New Guinea Institute of Medical Research; *LSTM* Liverpool School of Tropical Medicine; *ANC* antenatal clinic; *POC* point-of-care; *LM* light microscopy; *Y* yes; *N* no

Malaria transmission determined by *P.* *falciparum* prevalence by PCR in the study, or by EIR. Prevalence: Prevalence was determined as low transmission < 9%, moderate 9–25%, and high > 25%. Prevalence by EIR only for high transmission areas, > 5 per year. Samples size: all samples collected in study * study included 327 women, samples collected at multiple timepoints. Mol + sample indicates number of samples positive by molecular methods

The eight completed studies included all malaria endemic WHO regions except EMRO and transmission intensities ranging from low (n = 3, prevalence 4–7%) and medium (n = 1, prevalence 9.4%), to high (n = 4, prevalence > 25% or entomological inoculation rate (EIR) > 5 per year). Malaria transmission was determined by *P. falciparum* prevalence by PCR in the parent study, or by EIR. Prevalence was determined as low transmission < 9%, moderate 9–25%, and high > 25%. Prevalence by EIR was only used to determine high transmission areas. Samples were taken during regular ANC visits (7/8) and at delivery (3/8) and from symptomatic and asymptomatic individuals (Table 1). All studies compared the HS-RDT to a co-RDT and to a molecular test as a reference standard, though the type of co-RDT and molecular test used varied (Additional file 1: Table S1). The reference standards ranged in LOD from 0.02 to 6 parasites/μL. Two of the studies also quantified HRP2 concentrations present in the samples, using different methods [20, 21].

**Fig. 1:**
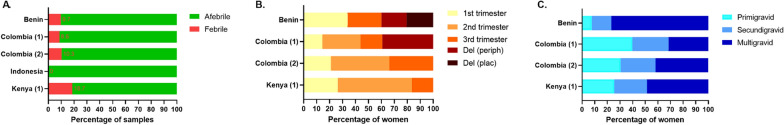
Descriptive of the population included in the HS-RDT evaluations. **A** Proportion of febrile samples. **B** Proportion of samples tested at each pregnancy time-point. **C** Proportion of primigravid (PG), secundigravid (SG) and multigravid (MG) women

Data sets for five of the eight completed studies were available and included in this review. The sample size of the five studies ranged from 270 to 942, with the Benin study [22] sampling the same women more than once (Table 1). At least 80% of women were asymptomatic in all studies, and in Indonesia the study excluded symptomatic women [23] (Fig. 1A). Febrile proportion data refers to the number of tests conducted in women with fever or a recent history of fever, divided by the total number of tests conducted. Most studies included women in all trimesters, except the study in Benin which sampled women only during 1st and 3rd trimesters and sampled the placenta at delivery (Fig. 1B). The Benin results are presented from the same women repeatedly tested at different timepoints and peripheral and placental results were included for the overall sensitivity estimations. All studies except Indonesia collected gravidity and trimester data of pregnant women. The study in Benin had a low percentage of primigravid women (7.7%; Fig. 1C). Although the Indonesian study did not provide details on the trimester or gravidity the gravidity in the parent study [24] was 27% primigravid, 30% secundigravid, and 43% multigravida. A significant difference for the Indonesia study is that samples were reconstructed from blood pellets and plasma.

The studies included high malaria infection prevalence areas (Benin [22] and Kenya [25] 18–36%), moderate (Indonesia 9% [23]) and low prevalence (Colombia 4–5% [26, 27]). The studies in low prevalence areas used reference standards with the lowest limits of detection (0.02–1 parasites/μL (p/μL)). The study in Indonesia (moderate transmission) had an intermediate prevalence in the overall study population of 9.4%, however a subset of these patients was selected for the HS-RDT evaluation study, hence the high PCR positivity rate of 58.5% from the study samples.

**Fig. 2:**
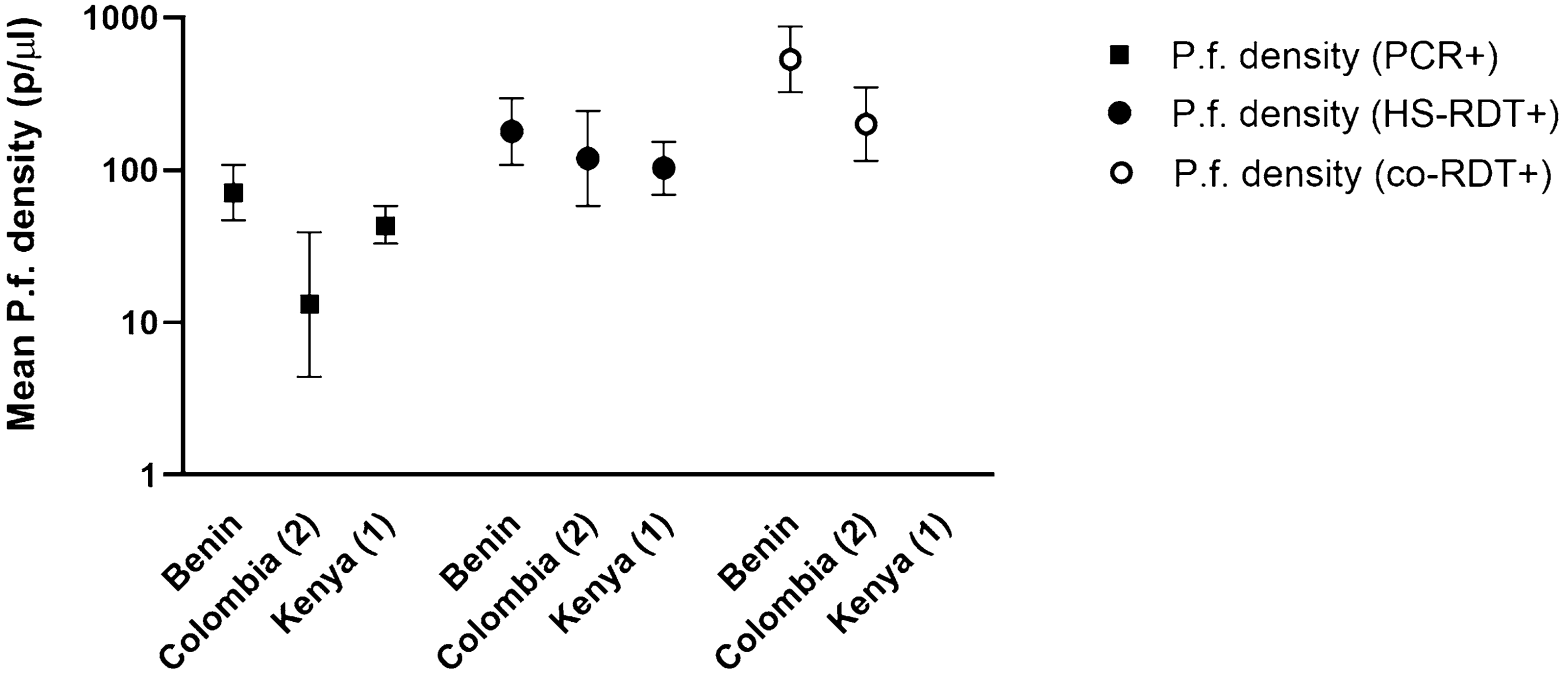
Geometric mean parasite densities and 95% CI of *P. falciparum* in samples positive by PCR or HS-RDT or co-RDT (for studies which calculated parasite density). The PCR Limit of Detection-Quantification reported by the different studies was: 2–5 p/ul (Benin), 0.02 p/ul (Colombia(2)), 3.2 p/ul (Kenya(1))*. *based on reference publication (not in-house estimation). Number of *P. falciparum* positive PCR and HS-RDT and co-RDT (when available) samples respectively per study: Benin = 179 and 153 and 76, Colombia (2) = 38 and 24 and 20, Kenya (1) = 172 and 107

Using molecular methods, the geometric mean parasitaemia, where available, ranged between 13 and 71 p/μL (Fig. 2). Mean parasite densities were lowest in the low transmission settings. While the geometric mean parasite density (GMPD) detected by molecular methods varied between studies, the GMPD of samples positive by HS-RDT was stable at around 100 p/μL (based on reference assay measurement) with no significant differences between studies. The lowest reported parasitaemias detected using HS-RDT were 2 and 5.14 p/μL in Benin and Kenya (1) studies, respectively. The GMPD detected by co-RDT was higher in both studies where these data were available (Benin 532 (95% CI 324–874) p/μL, and Colombia (2) 200 (95% CI 114–350) Fig. 2).

### Comparison of the performance of the HS-RDT and the co-RDT

**Fig. 3:**
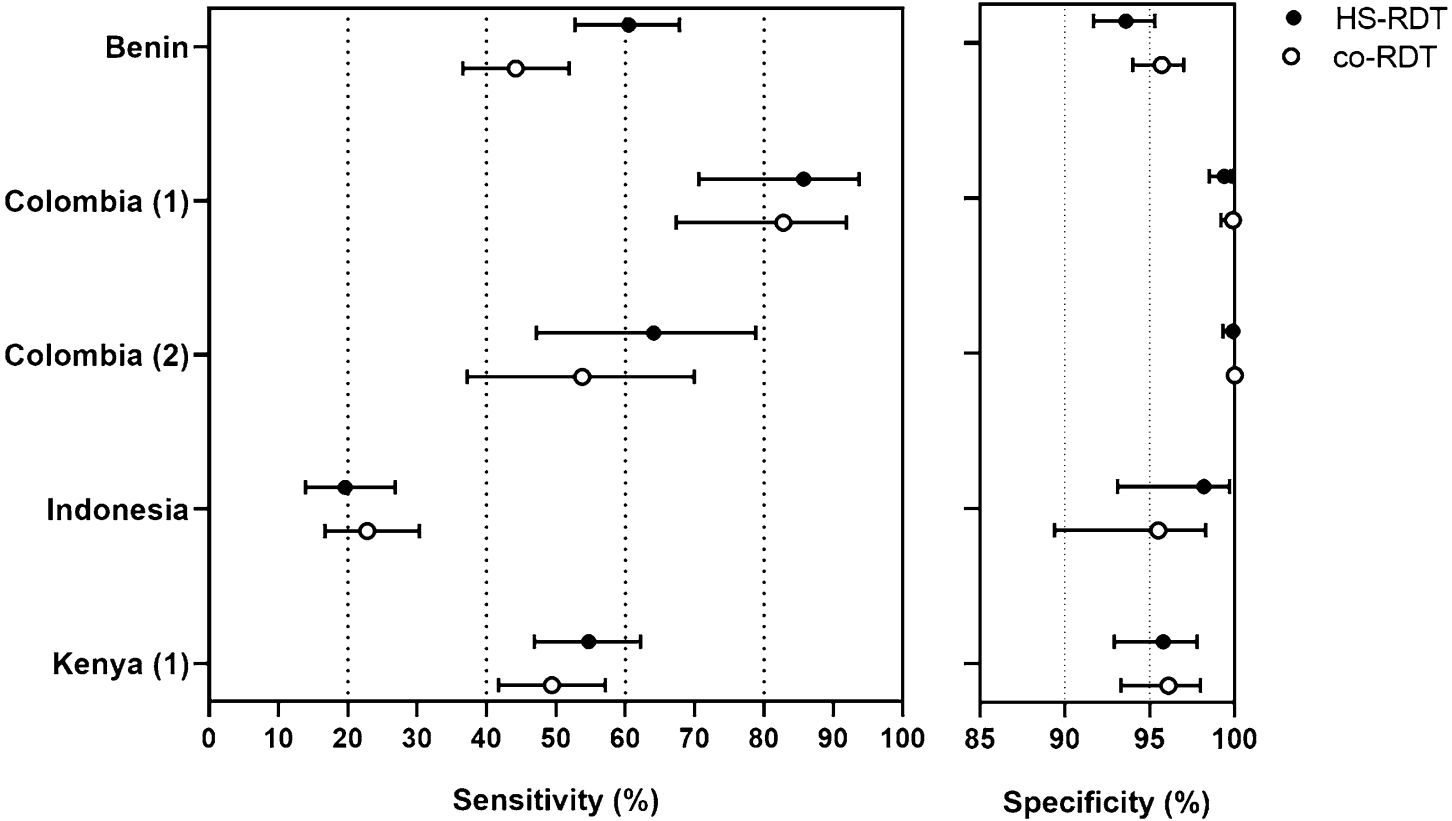
Sensitivity and specificity of the HS-RDT and the co-RDT for the detection of *P. falciparum* infection in pregnant women. The highly-sensitive rapid diagnostic test (HS-RDT: filled circles) was manufactured by Alere™, now Abbott. The conventional RDTs (co-RDT: unfilled circles) used in the different studies were manufactured by SD Bioline (Benin and Colombia (1)–(2)), Access Bio (Indonesia) and Premier Medical Corporation (Kenya (1)). Mean and 95% CI

Sensitivity, or the ability of the HS- and co-RDTs to detect true positives as determined by molecular testing, varied widely between studies from approximately 20 to 90% (Table 2; Fig. 3). In all studies the sensitivity of the co-RDT and HS-RDT were similar despite a wide difference between studies; while 4/5 of the studies showed a higher mean sensitivity of the HS-RDT compared to the co-RDT, in only one study was this a statistically significant difference (Table 2; Fig. 3). With the available data from three studies it is not possible to determine a relationship between sensitivity and parasite density (Additional file 1: Fig. S1). Specificity of both the HS-RDT and co-RDT was > 90% for all studies, with no significant difference between the HS-RDT and co-RDT (Table 2; Fig. 3). Heterogeneity between the studies was too great to allow a meta-analysis to be performed.

**Table 2:** Sensitivity and specificity of HS-RDT and co-RDT, and paired *t*-test comparisons in each study

Study	Sensitivity			Specificity			Parasite Density Geometric mean (0.95CI)			
	HS-RDT mean (0.95 CI)	Co-RDT mean (0.95 CI)	P value	HS-RDT mean (0.95 CI)	Co-RDT mean (0.95 CI)	P value	Reference method	HS-RDT + co-RDT -	HS-RDT	co-RDT
Benin	60.5 (52.7–67.8)	44.2 (36.9–51.9)	0.004	93.6 (91.7–95.3)	95.7 (94.0–97.0)	0.65	71.2 (47.0–107.9)	20.7 (10.8–39.6)	179.1 (108.3–296.2)	532.0 (324.0–874.0)
Colombia (1)	85.7 (70.6–93.7)	82.8 (67.3–91.9)	0.74	99.4 (98.5–99.8)	99.9 (99.2–100.0)	0.94				
Colombia (2)	64.1 (47.2–78.8)	53.8 (37.2–69.9)	0.38	99.9 (99.3–100.0)	100.0 (99.6–100.0)	0.99	13.2 (4.4–39.0)		119.2 (58.0–244.70)	200.1 (114.4–350.0)
Indonesia	19.6 (13.9–26.8)	22.8 (16.7–30.3)	0.51	98.2 (93.1–99.7)	95.5 (89.4–98.3)	0.50				
Kenya	54.7 (46.9–62.2)	49.4 (41.7–57.1)	0.34	95.8 (92.9–97.8)	96.1 (93.3–98.0)	0.94	43.0 (33.0–58.0)		103.0 (69.0–153.0)

### HS-RDT performance compared with co-RDT according to population and infection characteristics

**Fig. 4:**
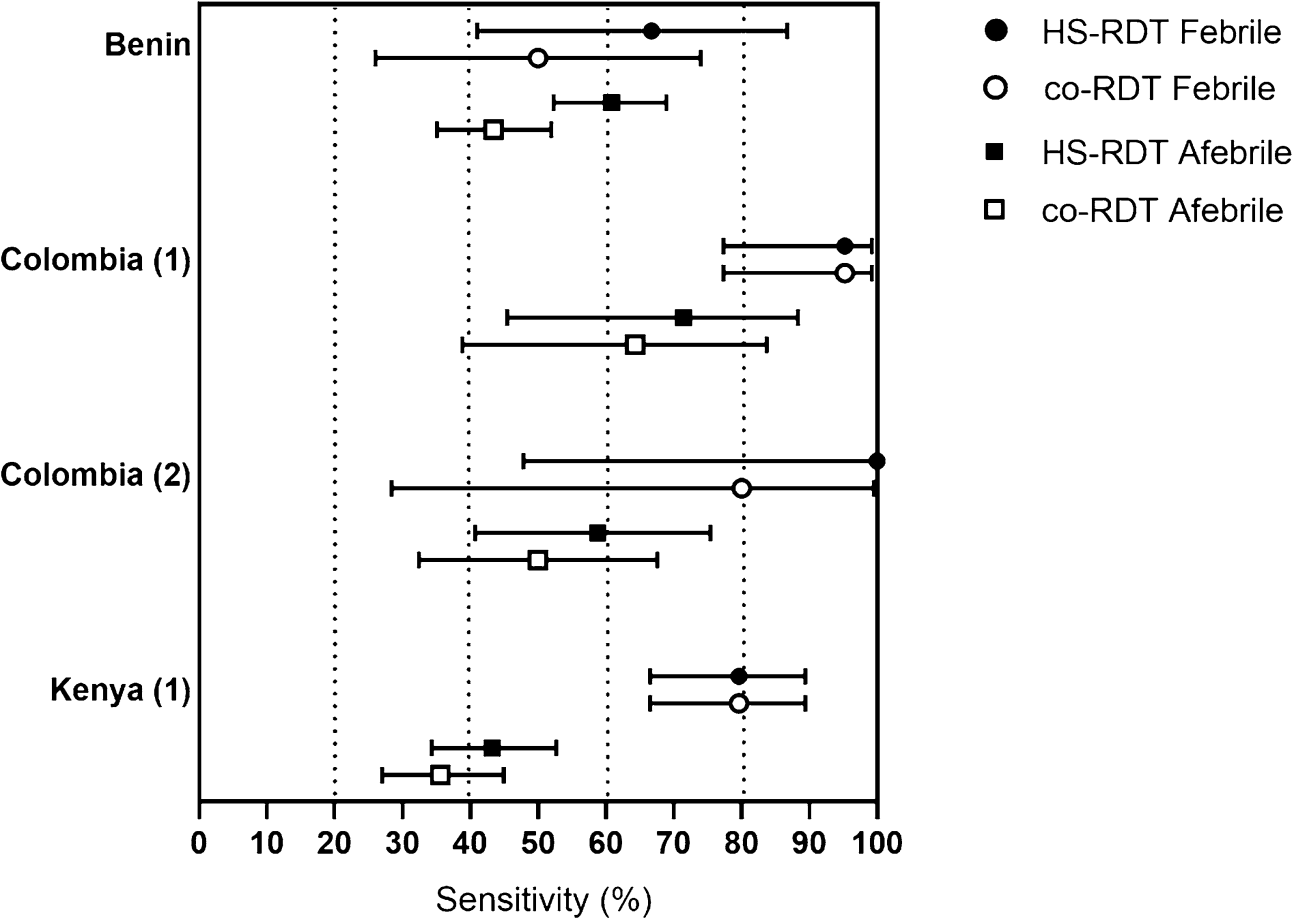
Sensitivity of the HS-RDT and the co-RDT for the detection of malaria by febrility. Samples from febrile patients in circles, afebrile patients in squares, mean and 95% CI

The HS-RDT was compared to the co-RDT to determine whether there were use cases where the HS-RDT could bring additional benefit. Sensitivity of both tests was higher in febrile compared to afebrile women as expected. In afebrile women, while 4/4 of the studies showed a higher mean sensitivity of the HS-RDT compared to the co-RDT, there was no statistical difference in sensitivity (Fig. 4).

**Fig. 5:**
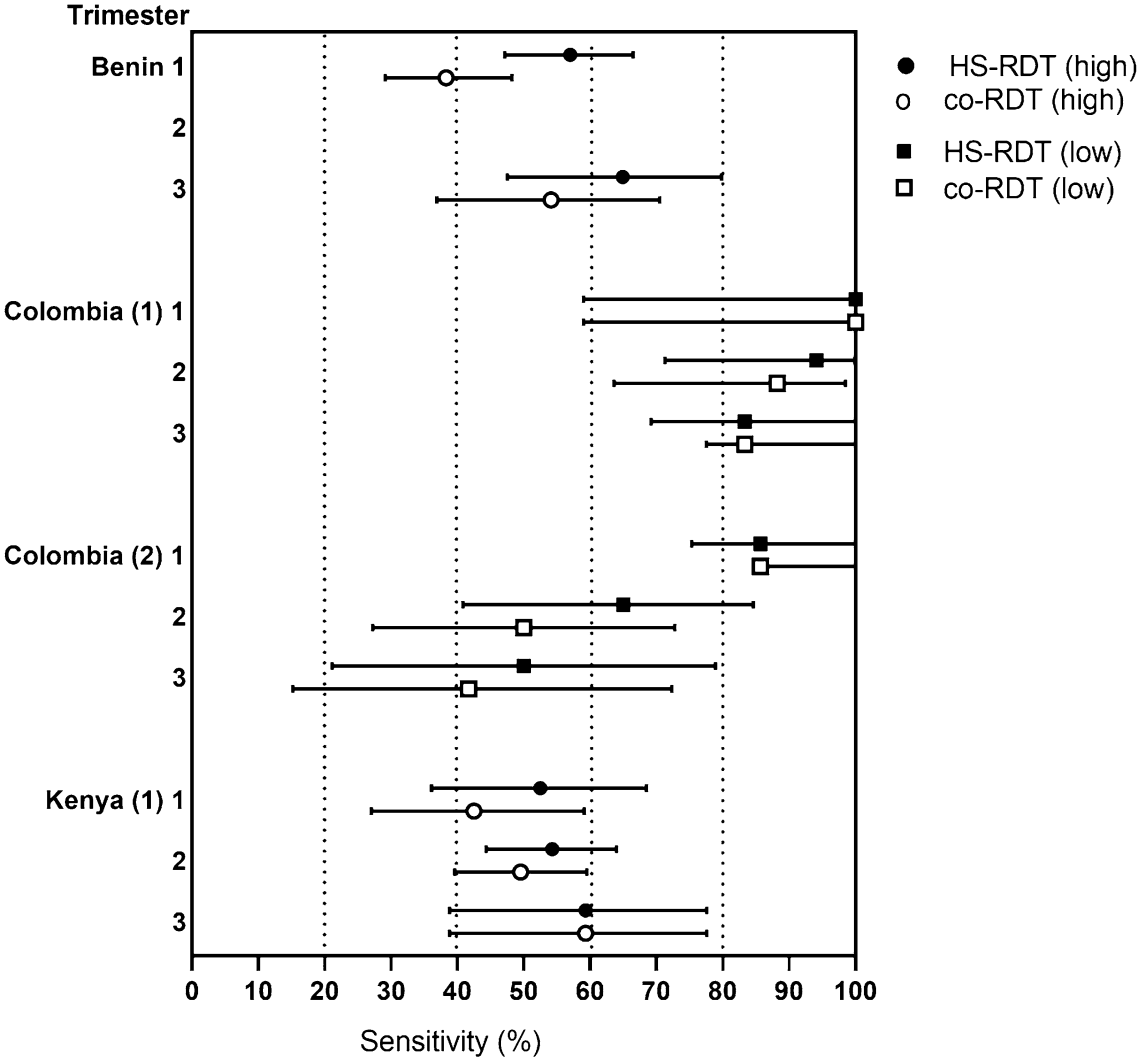
Sensitivity of the HS-RDT and the co-RDT for the detection of malaria by trimester of pregnancy in different transmission areas. Highly-sensitive rapid diagnostic test (HS-RDT); conventional RDT (co-RDT). Evaluations conducted in high-transmission settings (Benin and Kenya (1)) are represented by circles, and in low-transmission settings are represented by squares; black: HS-RDT; white; co-RDT. Mean and 95% CI

The sensitivity of the co-RDT and HS-RDT by pregnancy trimester was investigated in Fig. 5. In high transmission settings (Benin and Kenya) both RDTs appeared to be more sensitive in later trimesters, but this was not significant. In low transmission settings the reverse was observed with highest sensitivity in 1^st^ trimester, again there was no statistical difference.

**Fig. 6:**
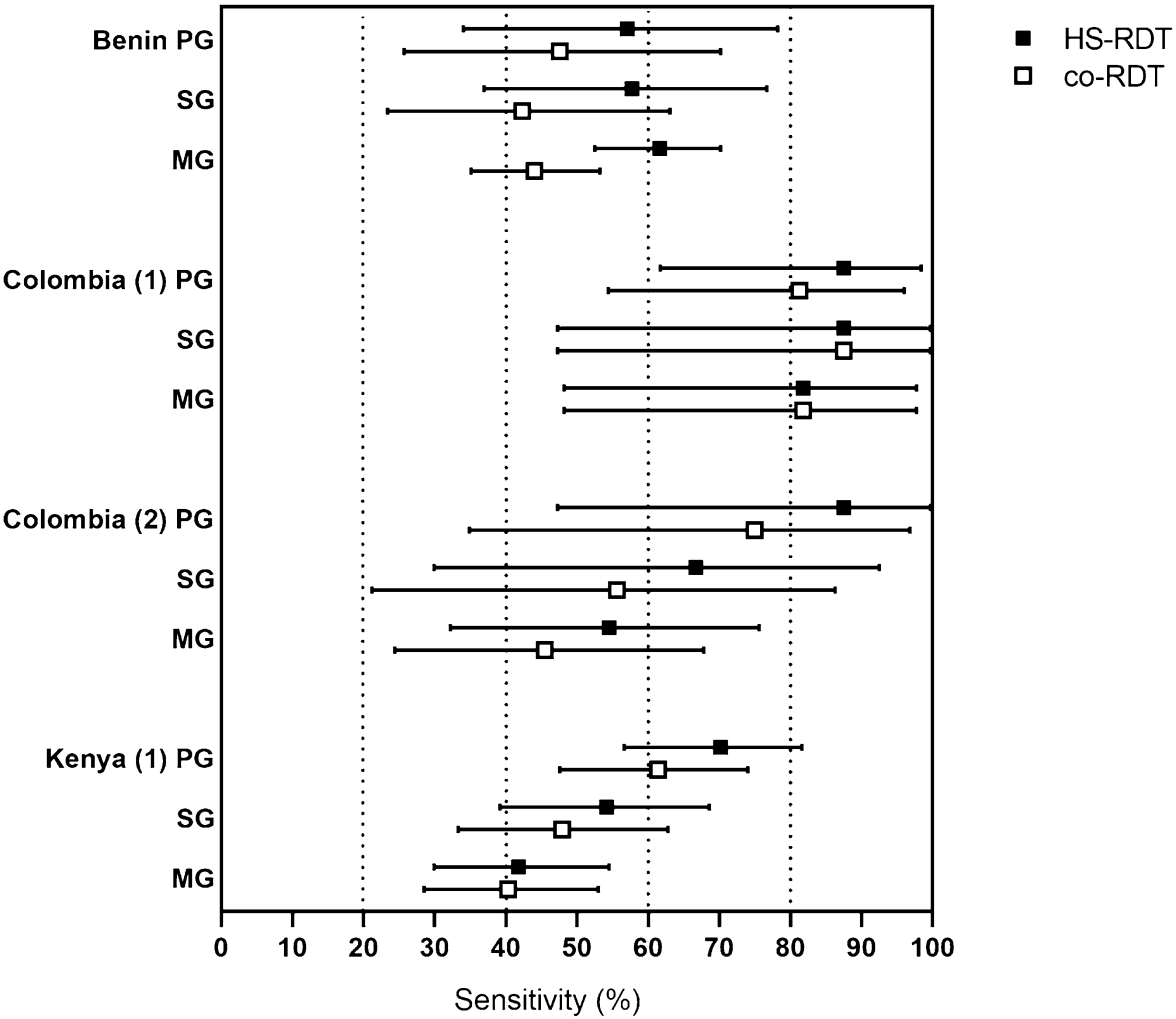
Sensitivity of the HS-RDT and the co-RDT for the detection of malaria by gravidity. Primigravid (PG), Secundigravid (SG), Multigravid (MG). Mean and 95% CI

There was no consistent pattern observed in sensitivity when compared by gravidity. The studies in Colombia (2) and Kenya showed decreasing sensitivity with increasing gravidity, and the study in Benin showed an increasing difference in sensitivity between the HS-RDT and co-RDT with increasing gravidity (Fig. 6).

HS-RDT sensitivity was not associated with *P. falciparum* prevalence reported in the studies (Spearman correlation r = − 0.314, p = 0.6), nor with the LOD of the reference method (Spearman correlation r = − 0.493, p = 0.3).

### Other evaluations of the use of HS-RDT for MiP

Clinical outcomes were investigated only in the Benin study, where the effect of the RDT used on maternal and birth outcomes was evaluated. The results from this single study investigating clinical outcomes indicated that infections detected with the HS-RDT but not with the co-RDT were associated with maternal anaemia, suggesting that testing based on the HS-RDT might be clinically relevant in this specific context and provide a direct health benefit for the pregnant women.

Different use cases were investigated in studies in Benin, Colombia and Malawi where the HS-RDT was used to detect placental infections from either placental or peripheral blood at delivery. The sensitivity of HS-RDT compared to co-RDT to detect infections from peripheral blood compared to placental blood was significantly higher in Benin, but both tests showed poor sensitivity in Colombia to detect parasites in placental blood (Additional file 1: Fig. S2). In the placental blood samples in Benin the HS-RDT detected double the number of positive samples compared to the qPCR reference standard (20 compared to 10). This could either be a low specificity of the HS-RDT in placental blood due to sustained presence of HRP2 following clearance of active infections or, the authors note, a low sensitivity of the qPCR in the placental blood due to possible inhibitors not present in the peripheral blood.

All studies identified the GMPD of samples which tested positive by HS-RDT at around 100p/µL (as calculated by extrapolation of qPCR Ct values or by microscopy). The study in Benin calculated the parasitaemia level at which samples were HS-RDT positive and co-RDT negative (N = 28) to have a mean density of 20.7 p/µL with HRP2 levels at a mean of 1049 pg/mL.

## Discussion

This paper describes the evidence landscape of HS-RDT performance compared with co-RDTs to diagnose *P. falciparum* malaria in pregnancy. In the reported studies HS-RDT does detect some very low parasitaemias, with the lowest density detected being ~ 2 parasite/μL. In the two studies with available data on parasitaemia the HS-RDT detected lower parasitaemias than the co-RDT. Overall, the detection of parasites is consistent across studies (~ 100 parasite/μL), therefore the variability in HS-RDT sensitivity is likely due to variations in epidemiological context such as parasite density distributions. Sensitivity of HS-RDTs ranged widely from 20 to 86% compared to molecular methods. The HS-RDT showed a slightly higher analytical sensitivity than the co-RDT in most studies but this difference was only statistically significant in the study from Benin.

The five studies with available data were heterogenous both geographically and by transmission intensity, aiding generalizability of the results. A key assumption is that molecular testing of peripheral blood can detect placental malaria, making these data comparable between sites [28]. Despite the limitations of using different PCR assays across studies infections in the low transmission sites tended towards lower parasite densities, supporting previous observations that there are more low-density infections in low transmission regions (< 100 parasite/μL [29, 30]). Walker et al. [8] showed that sensitivity decreased with decreasing parasite prevalence and hypothesized that HS-RDTs may have more impact than co-RDTs in low transmission areas with lower parasitaemias. The reviewed studies with the lowest transmission were in Colombia, and HS-RDTs were not significantly more sensitive than co-RDTs. However, looking at low density infections (parasite densities of ~ 10–100 p/μL), the HS-RDT identifies double the number of infections (26/44) as the co-RDT (13/44) in Benin [22], while in Colombia (2) the HS-RDT identifies 9/9 infections yet the co-RDT identifies only 6/9. This suggests an advantage to using HS-RDT to detect lower density infections.

Another source of variability in sensitivity is likely to be the samples used: although the RDTs are intended for use with fresh blood, the studies in Benin, Colombia (1), and Indonesia all used thawed blood, which may affect RDT performance. The study in Indonesia used thawed red blood cell pellets (stored: ~ 1 year), reconstituted with plasma to 30% haematocrit. This off-label use might account for the very low sensitivity of both the co-RDT and HS-RDT in this study. A further source of variation in sensitivity could be the reference standard used and its associated LOD. LODs ranged from ~ 0.02–6 parasite/μL. Across the five studies included in this analysis however, there was no trend for sensitivity to be associated with reference assay LOD.

Both RDTs tended to have higher sensitivity in febrile women compared with afebrile women, as expected. Prevalence of malaria infection is known to be highest at first ANC visit, and declines with gravidity by both PCR and co-RDT [4, 8]. The included studies did not show a clear relationship between HS-RDT sensitivity and gravidity, nor did this appear to be affected by transmission intensity. A recent modelling-based analysis [8] indicated that the main gain in sensitivity in using HS-RDTs among pregnant women should be expected when infections tend to have lower parasitaemias. When exploring this in the current review of limited studies, the clinical performance of the HS-RDT compared to co-RDTs only suggests a slight increase in analytical sensitivity in the 1st trimester in high-transmission settings, or the 2nd trimester in low transmission settings. One of the reasons behind this discrepancy is that the ‘better performing RDTs’ considered in these models are assumed to provide a 75% to 90% sensitivity, while most of the sensitivities observed in the HS-RDT field evaluations in the context of MiP fall below this range. The only study in which HS-RDT sensitivity significantly outperformed co-RDTs was in the first trimester in a high transmission setting (Benin) with a low proportion of primigravid women (8%). A major limitation of the analyses for febrility, gravidity and trimester are the low numbers of parasite positive women, limiting the statistical power of comparing sensitivities between the tests.

Another reason for minimal differences between HS-RDT and co-RDT could be that co-RDTs have a lower LOD than expected. While the criteria of detection of at least 200 parasite/μL is required for pre-qualification by WHO, the exact LOD is unknown and for some may approach the LOD of HS-RDT (i.e., they may detect lower numbers of parasites/uL). The SD Bioline Pf co-RDT was tested against the HS-RDT by Das et. al [14] and was found to be reactive to cultured parasites at ~ 49 parasite/µL compared to ~ 3.13 parasite/µL by the HS-RDT, i.e., in laboratory tests the LOD of this co-RDT was about four times more sensitive than the minimum required for WHO pre-qualification.

The main limitation of this evidence review is the difficulty of pooling data due to the heterogeneity of the studies, difference in reference assays and reporting formats, and small numbers of studies included. In turn, this heterogeneity is also a benefit to describe the potential advantages of the HS-RDTs in different situations. The main goal of diagnosing MiP is to reduce adverse clinical outcomes for women and their children. The study in Benin [22] identified that those infections in women that were detected with the HS-RDT but not by the co-RDT were more likely to suffer anaemia during pregnancy and suggested a higher risk of LBW, although this interaction is likely underpowered. It may also be due to the study design which treated any microscopy positive patients, which therefore identifies and treats those patients who would also be identified by co-RDT, while lower parasitaemic infections (i.e. those detected by HS-RDT) remain undetected and untreated which may lead to anaemia over time. The ongoing study in Burkino Faso [31] will investigate the impact of screening with HS-RDTs and treating women on placental malaria and LBW. These analyses of clinical outcomes are important as it is still unclear whether identifying more (low density) infections will make a difference to health outcomes. For example Rogerson et al. [9] identify four studies where infections detected by PCR but not by co-RDT were not associated with poorer outcomes in infants [32–35], and only one study in which infections detected by PCR and missed by microscopy were associated with LBW or prematurity [10], while a meta-analysis linked the presence of infections below co-RDT detection with clinical impacts [7]. More studies linking the use of more sensitive diagnostic tests with clinical outcomes are required.

Using HS-RDT in a similar manner to co-RDTs is supported, for example, using HS-RDT instead of co-RDT at ANC visits to assess community malaria transmission will provide more accurate prevalence estimates. However, other factors to consider in procurement include that HS-RDT has limited temperature stability and shelf-life claims compared to most co-RDT, and the costs of tests. Both tests will be limited in impact by the emergence of *hrp2/hrp3* deletion parasites that escape detection [30].

## Conclusion

Overall, the studies confirmed that the HS-RDT has a slightly higher analytical sensitivity than co-RDTs in various MiP epidemiological contexts. The use of the HS-RDT could be recommended in all cases where co-RDTs are currently used in ANC settings, although factors other than analytical sensitivity must be weighed in each context.

## Supplementary Information

**Additional file 1:** **Table S1.** Testing characteristics of the completed studies evaluating the use of the HS-RDT for the detection of MiP. **Table S2.** True and False positives and negatives by study. **Figure S1.** Sensitivity of the HS-RDT and parasite density of the maternal infections detected by the reference standard in each study. Densities represented as geometrical mean + confidence interval (p/μL). **Figure S2.** Sensitivity of HS-RDT at delivery in peripheral and placental blood.

## Data Availability

The data that support the findings of this study are available from the lead authors of the individual studies, but restrictions apply to the availability of these data, which were used under license for the current study, and so are not publicly available. Data are however available from the authors upon reasonable request.
