# Supplementary material for: Malaria in pregnancy (MiP) studies assessing the clinical performance of highly sensitive rapid diagnostic tests (HS-RDT) for Plasmodium falciparum detection
Source: Malar J. 2023 Feb 20;22:60. doi: 10.1186/s12936-023-04445-1 (PMC9942317; doi:10.1186/s12936-023-04445-1)
Supplement: Supplementary file 1 — Additional file 1: Table S1. Testing characteristics of the completed studies evaluating the use of the HS-RDT for the detection of MiP. Table S2. True and False positives and negatives by study. Figure S1. Sensitivity of the HS-RDT and parasite density of the maternal infections detected by the reference standard in each study. Densities represented as geometrical mean + confidence interval (p/μL). Figure S2. Sensitivity of HS-RDT at delivery in peripheral and placental blood. [file 12936_2023_4445_MOESM1_ESM.docx]

**Table S1:** Testing characteristics of the completed studies evaluating the use of the HS-RDT for the detection of MiP

| **n.** | **Country** | **Diagnostics (in addition to HS-RDT, NxTek™ Eliminate malaria Pf, Abbott Diagnostics)** | | | | | **Reference standard (for assessing performance)** | |
| --- | --- | --- | --- | --- | --- | --- | --- | --- |
|  |  | **Molecular diagnostic** | **LAMP** | **conventional RDT** | **Light Microscopy** | **HRP2 quantification** | **Ref. technique** | **Limit of Detection/Quantification (LoD/LoQ)** |
| ***Completed and analyzed*** | | | | | | | | |
| **1** | **Benin** | **qPCR** [41] | - | **SD BiolinePf** (SD – 05FK50) | **Yes** | **Yes** (Luminex)  [18] | qPCR | LoQ = 5 parasites / µL (2 parasites/ µL if positive) |
| **2** | **Colombia (1)** | **nPCR** [32] | - | **SD Bioline Pf** (SD – 05FK50) **SD Bioline Pf/Pv *** (SD – 05FK80) | **Yes** | **No** | nPCR | LoD = 1 parasite / µL |
| **3** | **Colombia (2)** | **qRT-PCR** [42] **nPCR** [32] | **LAMP-Pan/Pf** (Eiken) | **SD Bioline Pf/Pv** (SD – 05FK80) | **Yes** | **No** | qRT-PCR | LoD = 0.02 parasites / µL |
| **4** | **Indonesia** | **qPCR** [43,44] **nPCR** [32] | **LAMP-Pan** (Eiken) | **CareStart Pf/VOM Combo** (Access Bio – G0171) | **Yes** (not shown; none positive) | **No** | Composite: LAMP(Pan) + qPCR | *qPCR +LAMP:*  LoD = 2-5 parasites / µL *nPCR* (only in case of discordance):  LoD = 6 parasites / µL |
| **5** | **Kenya (1)** | **PET-PCR** [45] **qPCR** (not included  in the analysis) | **LAMP Illumigene**  (Meridian) | **First Response Pan/Pf** (Premier Medical Corporation - I16FRC) | **Yes** | **No** | PET-PCR | LoD = ~ 3.2 parasites / µL ** |
| ***Completed (analysis ongoing)*** | | | | | | | | |
| **6** | **Papua New Guinea** | **qPCR** [46] | **LAMP-Pan/Pf/Pv** (Eiken) | **CareStart Pf/PAN Combo** (Access Bio – G0131) | **Yes** | **No** | qPCR | LoQ = 0.33 parasites / µL (0.17 parasites/ µL if positive) |
| **7** | **Kenya (2)** | **qPCR** [43] | - | **CareStart Pf/VOM Combo** (Access Bio – G0171) | **Yes** | **No** | qPCR | LoD = 2-5 parasites / µL |
| **8** | **Malawi** | **RT-PCR** | - | **Paracheck Pf** (Orchid – 302030025) | **Yes** | **Yes** | RT-PCR | LoD = ~1 parasite / µL |

* Pf/Pv RDT used at point-of-care using fresh blood and Pf RDT used to test same stored samples as the ones analyzed by HS-RDT (frozen). The co-RDT and HS-RDT analysis presented in this article is based on the Pf test results. ** Based on the estimation provided in reference publication of the technique (not reported by the study authors)

Acronyms: qPCR, quantitative PCR ; qRT-PCR, quantitative Reverse Transcription PCR ; PET-PCR, Photo-induced-electron-transfer PCR ; nPCR, nested PCR ; LAMP, Loop-mediated isothermal DNA amplification ; LoD, Limit of detection ; LoQ, Limit of Quantification ; Pf, *Plasmodium falciparum* ; Pv, *Plasmodium vivax*

**Table S2:** True and False positives and negatives by study

|  | **HS-RDT** | | | | **Co-RDT** | | | |
| --- | --- | --- | --- | --- | --- | --- | --- | --- |
| **Study** | N True positives | N True negatives | N False positives | N False negatives | N True positives | N True negatives | N False positives | N False negatives |
| **Benin** | 103 | 721 | 49 | 69 | 76 | 737 | 33 | 96 |
| **Colombia (1)** | 30 | 698 | 4 | 5 | 29 | 701 | 1 | 6 |
| **Colombia (2)** | 25 | 818 | 1 | 14 | 21 | 819 | 0 | 18 |
| **Indonesia** | 31 | 110 | 2 | 127 | 36 | 107 | 5 | 122 |
| **Kenya** | 94 | 297 | 13 | 78 | 85 | 298 | 12 | 87 |

**
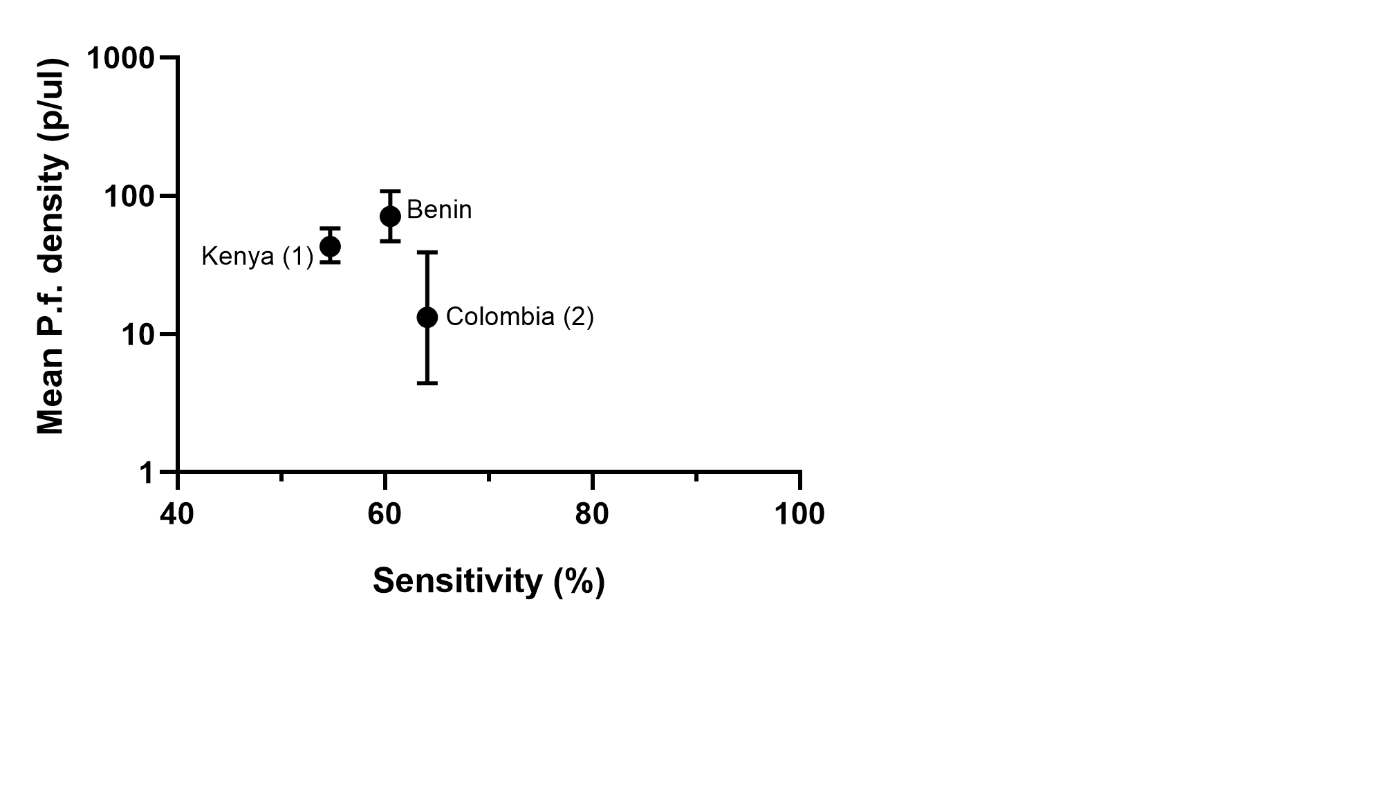
**

**Fig. S1** Sensitivity of the HS-RDT and parasite density of the maternal infections detected by the reference standard in each study. Densities represented as geometrical mean + confidence interval (p/μL).

**Fig. 2** Sensitivity of HS-RDT at delivery in peripheral and placental blood.

References

1. Diallo A, Ndam NT, Moussiliou A, Dos Santos S, Ndonky A, Borderon M, et al. Asymptomatic carriage of *Plasmodium* in urban Dakar: the risk of malaria should not be underestimated. PLoS ONE. 2012;7:e31100.
2. Seilie AM, Chang M, Hanron AE, Billman ZP, Stone BC, Zhou K, et al. Beyond blood smears: qualification of *Plasmodium* 18S rRNA as a biomarker for controlled human malaria infections. Am J Trop Med Hyg. 2019;100:1466–76.
3. Shokoples SE, Ndao M, Kowalewska-Grochowska K, Yanow SK. Multiplexed real-time PCR assay for discrimination of *Plasmodium* species with improved sensitivity for mixed infections. J Clin Microbiol. 2009;47:975–80.
4. Kamau E, Alemayehu S, Feghali KC, Saunders D, Ockenhouse CF. Multiplex qPCR for detection and absolute quantification of malaria. PLoS ONE. 2013;8:e71539.
5. Lucchi NW, Narayanan J, Karell MA, Xayavong M, Kariuki S, DaSilva AJ, et al. Molecular diagnosis of malaria by photo-induced electron transfer fluorogenic primers: PET-PCR. PLoS ONE. 2013;8:e56677.
6. Rosanas-Urgell A, Mueller D, Betuela I, Barnadas C, Iga J, Zimmerman PA, et al. Comparison of diagnostic methods for the detection and quantification of the four sympatric *Plasmodium* species in field samples from Papua New Guinea. Malar J. 2010;9:361.
